# Extragenic suppressor mutations in Δ*ripA* disrupt stability and function of LpxA

**DOI:** 10.1186/s12866-014-0336-x

**Published:** 2014-12-31

**Authors:** Cheryl N Miller, Shaun P Steele, Jason C Brunton, Ronald J Jenkins, Eric D LoVullo, Sharon A Taft-Benz, Artur Romanchuk, Corbin D Jones, Garry D Dotson, Edward J Collins, Thomas H Kawula

**Affiliations:** Department of Microbiology and Immunology, School of Medicine, University of North Carolina, Chapel Hill, NC 27599 USA; Department of Medicinal Chemistry, College of Pharmacy, University of Michigan, Ann Arbor, MI 48109 USA; Department of Biology, University of North Carolina, Chapel Hill, NC 27599 USA; Carolina Center for Genome Sciences, University of North Caroline, Chapel Hill, NC 27599 USA

**Keywords:** Extragenic suppressor mutations, Cell wall, Enzyme mutation, Enzyme mechanism, Membrane protein, RipA, *Francisella tularensis*, LpxA, Lipid A biosynthesis, UDP-N-acetylglucosamine

## Abstract

**Background:**

*Francisella tularensis* is a Gram-negative bacterium that infects hundreds of species including humans, and has evolved to grow efficiently within a plethora of cell types. RipA is a conserved membrane protein of *F. tularensis*, which is required for growth inside host cells. As a means to determine RipA function we isolated and mapped independent extragenic suppressor mutants in ∆*ripA* that restored growth in host cells. Each suppressor mutation mapped to one of two essential genes, *lpxA* or *glmU*, which are involved in lipid A synthesis. We repaired the suppressor mutation in *lpxA* (S102, LpxA T36N) and the mutation in *glmU* (S103, GlmU E57D), and demonstrated that each mutation was responsible for the suppressor phenotype in their respective strains. We hypothesize that the mutation in S102 altered the stability of LpxA, which can provide a clue to RipA function. LpxA is an UDP-N-acetylglucosamine acyltransferase that catalyzes the transfer of an acyl chain from acyl carrier protein (ACP) to UDP-N-acetylglucosamine (UDP-GlcNAc) to begin lipid A synthesis.

**Results:**

LpxA was more abundant in the presence of RipA. Induced expression of *lpxA* in the Δ*ripA* strain stopped bacterial division. The LpxA T36N S102 protein was less stable and therefore less abundant than wild type LpxA protein.

**Conclusion:**

These data suggest RipA functions to modulate lipid A synthesis in *F. tularensis* as a way to adapt to the host cell environment by interacting with LpxA*.*

**Electronic supplementary material:**

The online version of this article (doi:10.1186/s12866-014-0336-x) contains supplementary material, which is available to authorized users.

## Background

*Francisella tularensis* is a highly virulent Gram-negative bacterial pathogen that causes systemic infections in hundreds of animal species, including humans. Key pathogenic properties of *F. tularensis* are its ability to invade and replicate within many different host cell types and to suppress the initial host inflammatory response by innate immune cells. We previously identified a protein termed RipA, which is conserved among all sequenced *Francisella* species [1]. RipA is dispensable for growth in minimal media, but is required for *F. tularensis* virulence in mouse models of infection [1]. RipA is a homooligomeric cytoplasmic membrane protein that contains two cytoplasmic domains that are essential for RipA function [2]. The ∆*ripA* strain infects host cells and escapes the phagosome entering the cytoplasm similar to wild type *F. tularensis*, but fails to replicate within the cytosol [1]. Both transcription and translation of *ripA* are elevated at neutral pH coinciding with *F. tularensis* leaving the acidified vacuole and entering the neutral cytoplasm [3]. The *F. tularensis* ∆*ripA* strain stimulates a robust host inflammatory response activating the inflammasome and MAPK pathways, a response that is not seen with wild type *F. tularensis* [4]. In *Francisella novicida*, ∆*ripA* activates increased levels of AIM2-dependent pyroptosis, suggesting the bacterial membrane is compromised [5]. Determining RipA function would provide an important insight into the specific mechanisms used by *F. tularensis* to grow within eukaryotic cells. Given the scant information obtained from bioinformatics analysis of RipA, and the eclectic group of organisms that express RipA-like proteins [2], we took genetic and biochemical approaches to determine RipA function. We isolated independent extragenic suppressor mutations of ∆*ripA* that restored intracellular growth, and all suppressor mutations mapped to either *lpxA* or *glmU*. Both *lpxA* and *glmU* encode essential enzymes involved in lipid A synthesis [6,7]. Repairing the mutation in *lpxA* or *glmU* abrogated the suppressor phenotype supporting that the mutation in *lpxA* and *glmU* were responsible for the suppressor phenotype in their respective strains. LpxA is an essential UDP-N-acetylglucosamine acyltransferase that catalyzes the transfer of an acyl chain from acyl carrier protein (ACP) to UDP-N-acetylglucosamine (UDP-GlcNAc) to begin lipid A synthesis, which is the hydrophobic anchor of lipopolysaccharide (LPS) [8]. The active site and substrate binding pocket of LpxA is well characterized in *Escherichia coli* [9]. LpxA forms a trimer composed of three identical subunits with each subunit composed of two domains [10]. The N- terminal domain forms a left-handed helix of short parallel β-sheets and the C-terminal domain is composed of four α-helices. The active site in LpxA is located in a cleft between the two subunits, and the amino acids essential for LpxA function in *E. coli* are K76, H122, H125, H144, M156, A158, H160, V171, G173, and R204 [9,11]. *E. coli* LpxA has a strict preference for hydroxymyristoyl (C:14) fatty acids, and this preference is referred to as a hydrocarbon ruler [9]. LpxA from *F. tularensis* shares 44% identity and 66% similarity with *E. coli* LpxA, but does not share the same preference for C:14 fatty acids [12]. Instead, *F. tularensis* LpxA has evolved a relaxed acyl chain specificity and can attach longer fatty acids onto the 3′ hydroxyl acyl of UDP-GlcNAc: incorporating either hydroxystearoyl (3-OH C18:0), or hydroxypalmitoyl (3-OH C16:0) fatty acids. GlmU is also involved in the synthesis of lipid A. GlmU is an essential bifunctional acetyltransferase and an uridyltransferase that performs the last two sequential steps in the synthesis of UDP-GlcNAc. UDP-GlcNAc is an important component of both lipid A, and peptidoglycan.

Herein we describe the isolation and characterization of an extragenic suppressor mutation of ∆*ripA* in *F. tularensis* subspecies *holarctica* live vaccine strain (LVS) that mapped to *lpxA* and restored intracellular growth*.* We show that RipA and LpxA co-immunoprecipitate*.* Induced expression of *lpxA* in the absence of *ripA* is detrimental to bacterial growth, and the suppressor mutation LpxA T36N renders the protein less stable. In summary, *F. tularensis* acquires extragenic suppressor mutations in *lpxA* to restore intracellular growth in the absence of *ripA*.

## Results and discussion

### Extragenic suppressor mutation enrichment in the ∆*ripA* background

To enrich for extragenic suppressor mutations in the ∆*ripA* strain we performed several rounds of infections lasting 36 hours in J774A.1 cells whereby the bacteria isolated from the first round of infections were then used to inoculate a new flask of J774A.1 cells. Using the repeated rounds of infections in host cells, an independently derived extragenic suppressor of the ∆*ripA* strain was isolated, named S102. Using whole genome sequencing with the Genome Analyzer IIx (Illumina) we mapped the suppressor mutation in S102 by conducting comparative analysis aligning the sequencing reads from the suppressor mutant strain to the annotated genome on NCBI (NC_007880) (Table 1). The sequencing results were remarkably similar to the published annotated genome, with only 4 polymorphisms detected and 55 zero coverage regions detected (Table 1). Of the 55 zero coverage regions, 54 mapped to insertion sequence (IS) elements. IS elements have small inverted repeats, where the short sequence reads cannot span the entire length of the conserved repeated sequence, allowing the reads to align to incorrect IS elements in the genome (Additional file 7: Table S2). The LVS genome contains 59 ISFtu1 elements and 43 ISFtu2 elements that range in size from about 100 bp to about 900 bp. The remaining zero coverage region was the *ripA* (FTL_1914) locus, confirming that the extragenic suppressor mutation was in a Δ*ripA* background.

**Table 1:** **Polymorphisms detected by whole genome sequencing**

**Gene**	**Name**	**Amino acid**	**Location**	**Polymorphism verified**
**FTL_0146**	ABC transporter ATP binding protein	R341L	152667	wt, Δ*ripA*, and S102
			1023 bp from start	
**FTL_0539 (** ***lpxA*** **)**	UDP-N-acetylglucosamine acyltransferase	T36N	522331	Unique to S102
			107 bp from start	
**FTL_0717 (** ***rne*** **)**	Ribonuclease E	K38T	709489	Δ*ripA*, and S102
			114 bp from start	
**FTL_1388 (** ***nadB*** **)**	L-aspartate oxidase	A93T	1317802	wt, Δ*ripA*, and S102
			278 bp from start	

To determine if the mutations identified by whole genome sequencing were unique to S102 and not just variations present in our laboratory strain, the regions of interest were PCR amplified and analyzed using wild type *F. tularensis*, Δ*ripA* and Δ*ripA* suppressor genomic DNA. All the strains tested had two polymorphisms, confirming that the laboratory strain is similar but not identical in sequence to the annotated genome online (NC_007880) (Table 1). The first polymorphism was a base pair change in FTL_0146, an ABC transporter ATP-binding protein that caused an R341L missense mutation. The second polymorphism was a point mutation in FTL_1388, a L-aspartate oxidase, leading to the missense A93T mutation. The Δ*ripA* strain contained an additional missense mutation in FTL_0717, ribonuclease E, K38T. The missense mutation in ribonuclease E does not contribute the growth defect of the Δ*ripA* strain, because the growth defect in this, and all other Δ*ripA* strains, was restored by complementation with wild type *ripA* alone (data not shown).

The only unique mutation found in the suppressor strain S102, was located within FTL_0539, which encodes LpxA (Table 1). *lpxA* is located in an essential operon containing five genes involved in membrane synthesis. LpxA is an acyltransferase that catalyzes the first step in lipid A biosynthesis, using UDP-N-acetylglucosamine (UDP-GlcNAc) and acyl-ACP to synthesize the anchor of lipopolysaccharide. The polymorphism within *lpxA* was a point mutation leading to the substitution T36N. The amino acid substitution was located within the hexapeptide transferase domain close to the N-terminus, and was not within the active pocket. Thus, the location of the mutation is unlikely to alter the active pocket but could alter protein stability.

**Figure 1:**
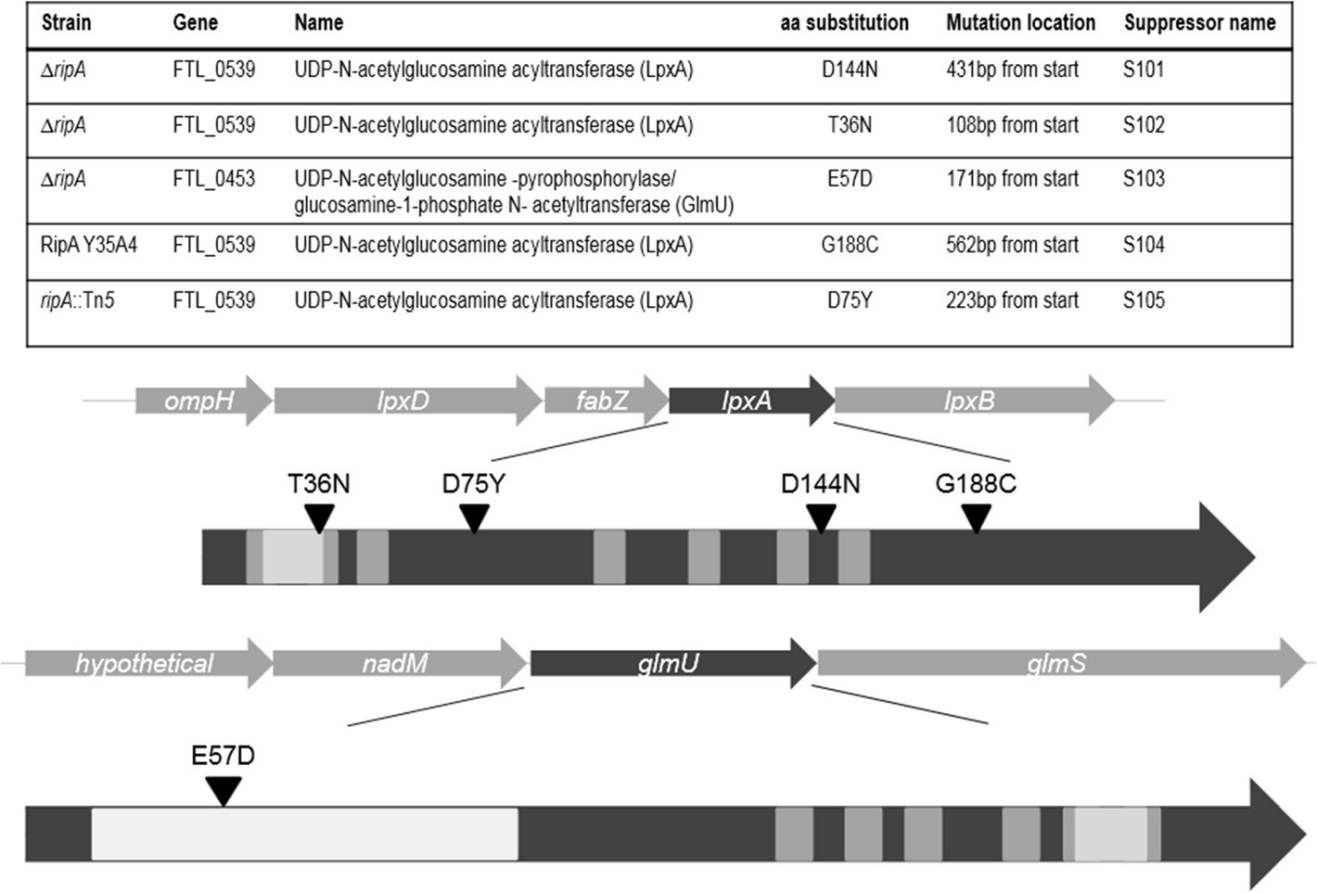
**Extragenic suppressor mutations map to** ***lpxA*** **and** ***glmU*** **.** Four of the five independently derived extragenic suppressor mutations mapped to *lpxA. lpxA* is centrally located in an essential operon containing five genes. The amino acid substitutions in LpxA were mainly located along the left-handed helix of the short parallel β-sheet. LpxA contains hexapeptide repeats that are a signature of an acetyltransferase enzyme (shown in gray). One extragenic suppressor mutation mapped to *glmU*, which is also centrally located in an essential operon containing 4 genes. The amino acid substitution mapped within the uridyltransferase domain located close to the N-terminus. LpxA and GlmU are both involved in membrane synthesis, and both proteins contain hexapeptide transferase signature domains.

**Figure 2:**
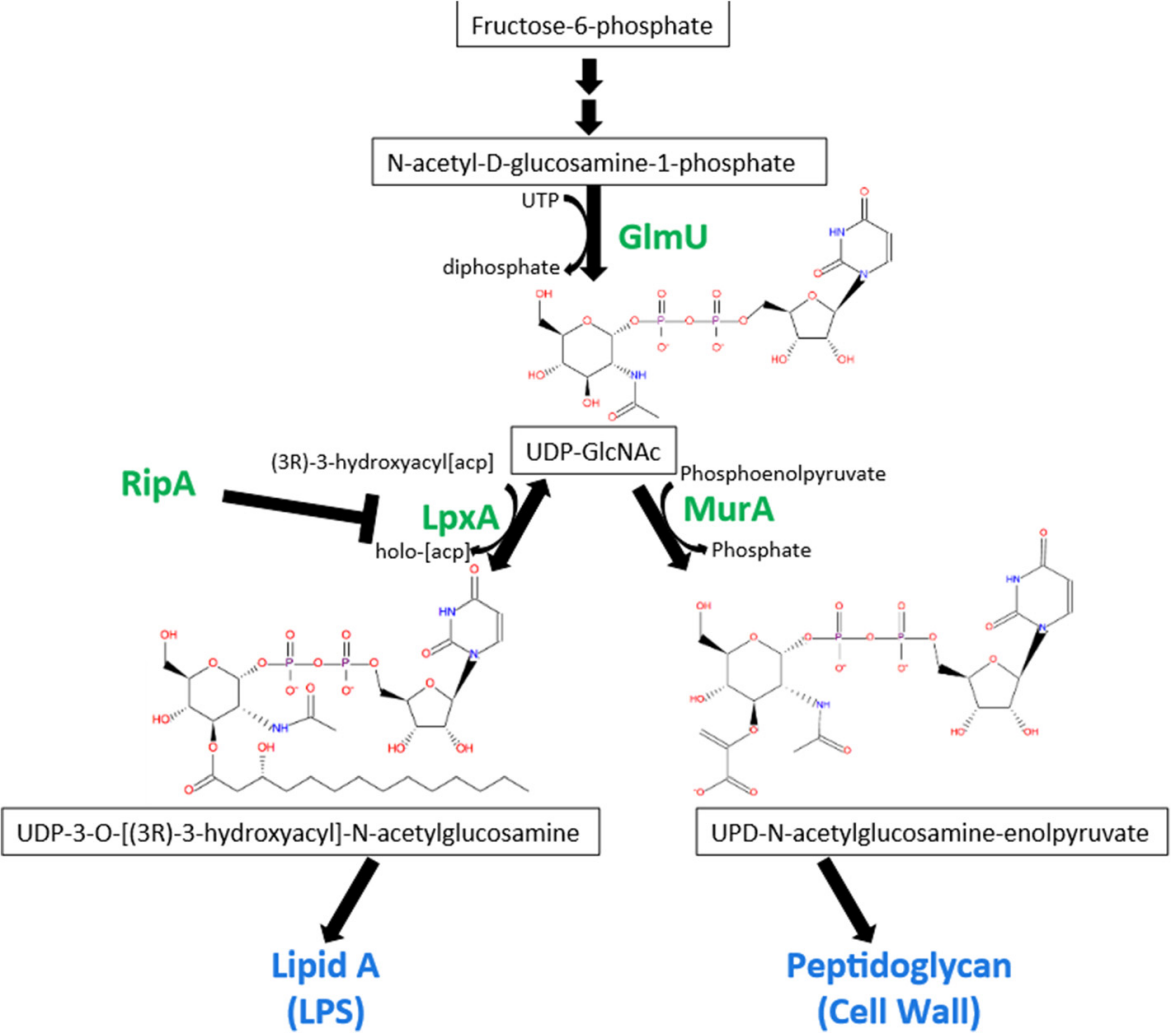
**The biosynthetic pathway of LpxA and GlmU.** UDP-N-acetylglucosamine (UDP-GlcNAc) is synthesized from fructose-6-phosphate with the last two sequential steps synthesized by the bifunctional acetyltransferase and uridyltransferase, GlmU. The substrate UDP-glucosamine is either used by LpxA in conjunction with acyl-ACP in a reversible reaction to make a precursor molecule of lipid A, or is used by MurA to make a precursor molecule of the cell wall.

By sequencing more Δ*ripA* strains with recovered intracellular growth, three additional independently derived extragenic suppressor mutations also mapped to *lpxA,* and one mapped to *glmU.* The mutations in *lpxA* resulted in amino acid substitutions located primarily along the left-handed helix of the short parallel β-sheet of LpxA (Figure 1). LpxA and GlmU are both involved in the synthesis of lipid A. GlmU is an essential acetyltransferase and an uridyltransferase that performs the last two sequential steps in the synthesis of UDP-GlcNAc. UDP-GlcNAc is an important component of both lipid A, and peptidoglycan (Figure 2). The missense mutation identified in *glmU* resulted in a substitution E57D in the uridyltransferase domain.

### LpxA T36N and GlmU E57D rescued growth of ∆*ripA* in eukaryotic cells

**Figure 3:**
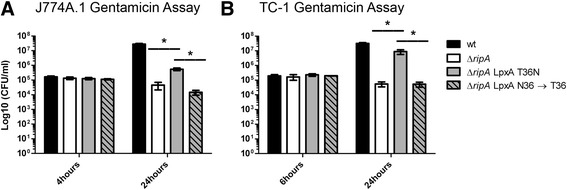
**The S102 extragenic suppressor mutant phenotype and repair in J774A.1 and TC-1 cells.** Intracellular replication of wild type *F. tularensis* LVS, ∆*ripA,* S102 (T36N), and the strain containing the repaired S102 extragenic suppressor mutation (LpxA N36 → T) was assessed within J774A.1 macrophages **(A)** and TC-1 epithelial cells **(B)** using the gentamicin protection assay. Each graph is the mean of at least three independent experiments done in triplicate, and the error bars represent the standard deviation. Statistical significance was determined using Student’s *t* tests comparing LpxA T36N to ∆*ripA* or to LpxA N36 → T36. *, *P* < 0.05.

To determine if the unique mutation found in *lpxA* was responsible for the suppressor phenotype, we repaired S102 *lpxA* base pair mutation, changing adenine 107 back to wild type cytosine, resulting in the reversion of T36N to N36T. S102 proliferated in 24 hours in both J774A.1 and TC-1 cells (Figure 3A and B). The repaired *lpxA* abrogated the suppressor growth phenotype in both J774A.1 and TC-1 cells demonstrating that the single point mutation in *lpxA* was responsible for the suppressor phenotype (Figure 3A and B). Repairing the suppressor mutation in *glmU* also abrogated the suppressor growth phenotype in both J774A.1 and TC-1 cells demonstrating the single mutation in *glmU* was responsible for the suppressor phenotype in S103 (Additional file 1: Figure S1A and B).

### LpxA and RipA co-immunoprecipitate

**Figure 4:**
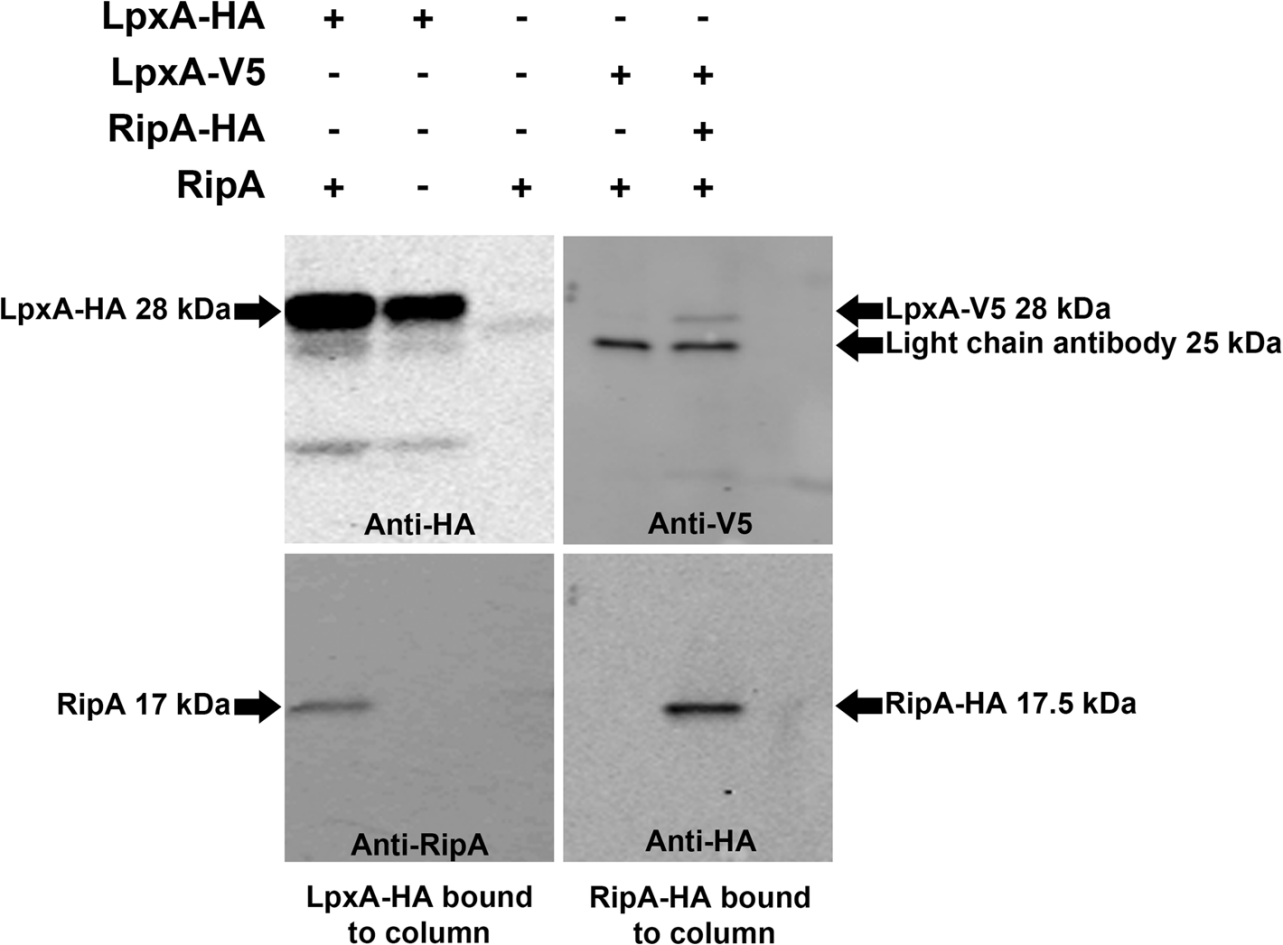
**LpxA and RipA co-immunoprecipitate.** The left two Western blots demonstrate LpxA-HA binds and pulls down RipA from *F. tularensis* whole cell lysates. The Western blots on the left were probed with primary antibodies to anti-HA and anti-N-terminal RipAaa1-19. The right two Western blots demonstrate that RipA-HA binds and pulls down LpxA-V5 from *F. tularensis* whole cell lystates. Western blots on the right were probed with primary antibodies to anti-HA and anti-V5. This figure is a representative of at least three independent experiments.

RipA is a cytoplasmic membrane protein containing two domains that are essential for function and are accessible to the cytoplasm [2]. LpxA is a soluble protein that interacts with acyl-ACP on the cytoplasmic membrane. To evaluate interactions between RipA and LpxA we performed co-immunoprecipitation reactions using *F. tularensis* lysates. The first precipitation reaction was performed with a *F. tularensis* strain containing the wild type copy of *ripA* in the chromosome and *lpxA-HA* expressed from a plasmid. RipA immunoprecipitated with LpxA-HA on an anti-HA agarose column (Figure 4). The reciprocal co-immunoprecipitation was performed with *ripA-HA* integrated into the chromosome and *lpxA-V5* expressed from a plasmid. In both conditions LpxA and RipA co-immunoprecipitated (Figure 4). Given the possibility that RipA and LpxA may interact, we next wanted to determine if the localization of LpxA was altered in the Δ*ripA* strain. Membrane fractions were separated using ultracentrifugation and Sarkosyl extraction for the strain containing *lpxA-HA* on a plasmid. The soluble, inner membrane, and outer membrane fractions were normalized to equivalent total protein and analyzed by Western blot. LpxA-HA was present in the inner membrane fraction in both the wild type *F. tularensis* stain and the Δ*ripA* strain, suggesting localization of LpxA was not influenced by the presence or absence of RipA (Additional file 2: Figure S2).

### The extragenic suppressor mutation in *lpxA* does not alter membrane composition and LPS

There is substantial diversity among bacterial fatty acids profiles and *F. tularensis* is unique for its relative abundance of long chain saturated and monoenoic acids [13]. *F. tularensis* LVS has a lipid content of 21% by dry weight, and the two major phospholipid components are phosphatidylethanolamine (PE; 76%) and phosphatidylglycerol (PG; 24%) [14]. In *F. tularensis* PE contains a high proportion of 24:0 fatty acids, while PG contains mainly C18:0 and C22:0 fatty acids. Another unique property of the membrane composition of *F. tularensis* is the abundant amount of free lipid A present on the outer membrane of the bacteria that is not attached to O-antigen sugars [15]. In *F. novicida* two forms of free lipid A were identified: one containing a galactosamine sugar attached to the 1′ phosphate position (compound A1), and the other containing a glucose sugar attached to the 6′ OH group on glucosamine (compound A2) [15]. We analyzed the lipid profile of purified membrane fractions from wild type *F. tularensis*, Δ*ripA* and S102 in order to identify any differences in the membrane composition between the strains. As reported previously, the most abundant components of the purified membrane fractions were PE, PG, phosphatidylcholine (PC), and lipid A (Additional file 3: Figure S3A) [15]. We also observed similar quantities of free lipid A in *F. tularensis* LVS as seen in *F. novicida,* however no compound A2 was detected corresponding with a glucose sugar linked to the 6′ position of lipid A, confirming previous findings [15,16]. In all broth grown strains analyzed, there was a similar composition of lipids as determined by thin layer chromatography (TLC) (Additional file 3: Figure S3A). Others have shown that *F. tularensis* membrane profiles changed during intracellular growth [17]. To determine if RipA is involved in modifying the membrane during host infection, we purified bacterial membrane fractions from J774A.1 cells infected with either wild type *F. tularensis* LVS or Δ*ripA* and subsequently analyzed the lipid profiles by TLC. We observed changes in the membrane composition of *F. tularensis* inside J774A.1 cells as compared to broth grown organisms, but the changes were not influenced by the absence of *ripA* (Additional file 3: Figure S3B). In addition, there were no consistent changes among the LPS banding pattern observed by Western blot (Additional file 3: Figure S3C), or in LPS quantity of wild type *F. tularensis*, Δ*ripA*, and S102 measured by the purpald assay (Additional file 4: Figure S4) [18]. Nor where there any changes in the lipid A profile between the different strains when analyzed by mass spectrometry (Additional file 5: Figure S5).

### LpxA protein levels are significantly lower in the Δ*ripA* strain compared to wild type

**Figure 5:**
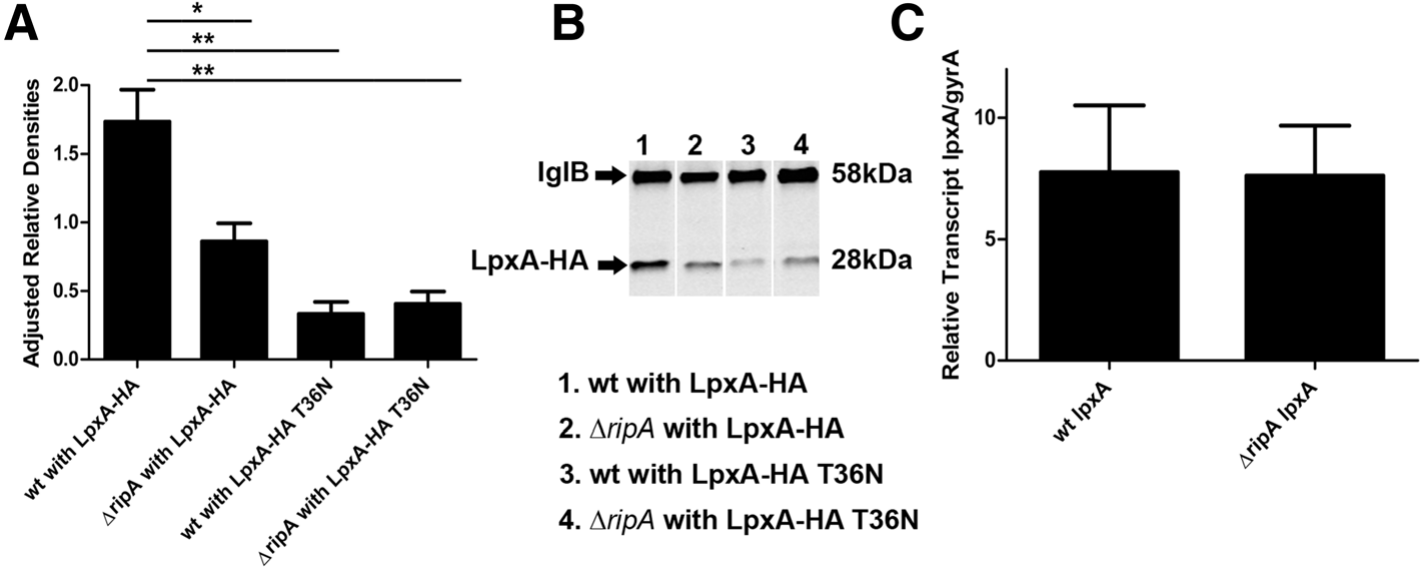
**RipA is necessary for stability of LpxA. (A)** Relative protein concentrations were determined from mid log phase cultures treated with 1.75 mg ml^−1^ chloramphenicol for an hour to stop protein translation. The relative abundance of LpxA protein was quantified using Image J with IglB as a loading control for equal protein from three independent experiments. Statistical significance was determined by comparing LpxA-HA adjusted relative protein values for wild type to each of the respective mutants. *, *P* < 0.05; **, *P* < 0.005. **(B)** Representative Western blot probed for LpxA-HA and IglB protein loaded with equal protein. **(C)** Transcript levels of *lpxA* in wild type *F. tularensis* and the ∆*ripA* strain measured by qRT-PCR. Data represent the relative transcript of *lpxA* normalized to *gyrA* from three independent experiments done in triplicate and the relative transcripts were not statistically different.

A potential consequence of RipA-LpxA interaction is that RipA may stabilize LpxA. To determine if RipA influences the stability of LpxA we first quantified the amount of LpxA protein present in wild type *F. tularensis* and the ∆*ripA* strain. LpxA protein levels were 2-fold higher in wild type *F. tularensis* than in ∆*ripA,* when normalized to the loading control IglB after chloramphenicol treatment (Figure 5A and B). The LpxA-HA T36N protein levels were low in both wild type and ∆*ripA*, suggesting that the suppressor mutation in LpxA may reduce protein stability (Figure 5A and B). To further analyze whether the observed differences in LpxA protein levels were due to protein stability or changes in transcription, we quantified *lpxA* mRNA in wild type and ∆*ripA* strains*.* We performed quantitative real time PCR on *lpxA* and normalized transcript levels to the housekeeping gene *gyrA*. The relative transcript levels of *lpxA* were not significantly different between wild type *F. tularensis* and ∆*ripA* indicating that the regulation of *lpxA* is posttranscriptional (Figure 5C).

### Inducing expression of LpxA negatively affects the ∆*ripA* strain

**Figure 6:**
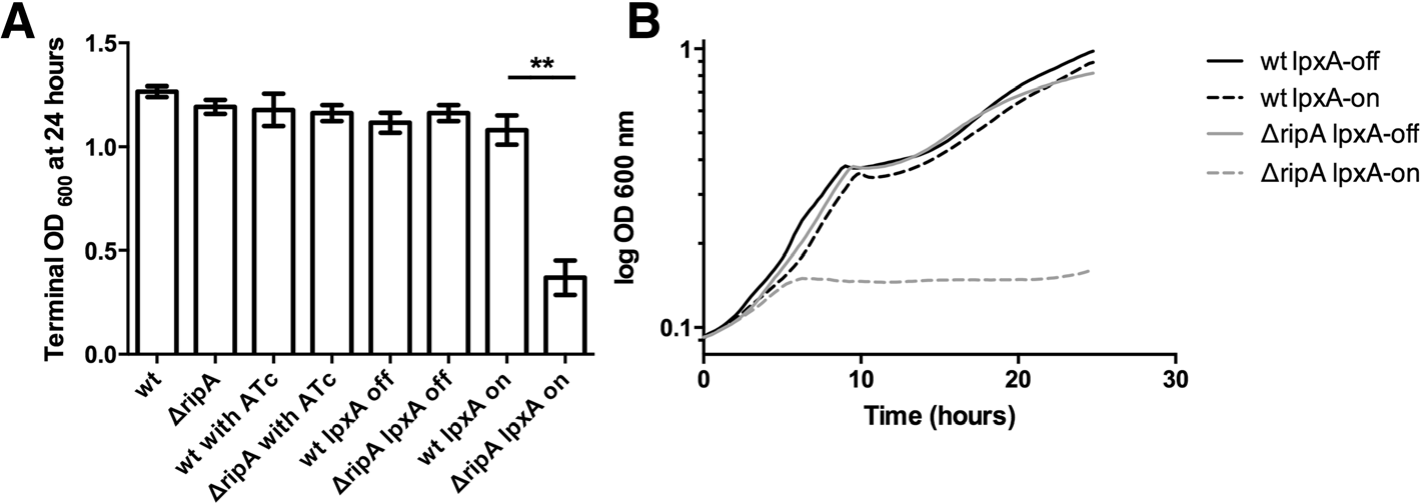
**Inducing expression of lpxA stops growth of the ∆** ***ripA*** **strain. (A)** Terminal OD_600_ of strains grown in CDM with or without 100 ng ml^−1^ of anhydrotetracycline (ATc) at 24 hours. LVS, ∆*ripA,* LVS containing *lpxA* in trans, and ∆*ripA* containing *lpxA* in trans. ATc was used to induce expression of desired genes under the control of the *rpsLp-tetR* system [19]*.* **(B)** Representative growth curve in CDM with induced expression of *lpxA* or *ripA* using 100 ng ml^−1^ of ATc. Terminal OD_600_ at 24 hours of S102 and S102 with *ripA* in trans, under the control of the *rpsLp-tetR* system. Representative growth curve in CDM where *ripA* expression was induced with 100 ng ml^−1^ of ATc. The growth curves were repeated at least three times and statistical significance was determined using Student’s *t* tests. **P < 0.001.

Elevated levels of LpxA protein in the absence of RipA could be detrimental for bacterial growth and therefore responsible for the failure of the Δ*ripA* strain to grow *in vivo*. To test if LpxA activity and the flux of UDP-GlcNAc and acyl-ACP are important for bacterial growth we created an anhydrotetracycline (ATc) inducible expression system for *lpxA* to control the amount of LpxA present in both wild type *F. tularensis* and the ∆*ripA* strain [19]. When *lpxA* expression was induced in broth the ∆*ripA* strain stopped growing (Figure 6A and B), whereas, growth of wild type *F. tularensis* was barely affected by the increased expression of *lpxA*. These results suggest that activity and amount of LpxA is very tightly controlled and is affected by RipA, which may be important for *F. tularensis* to adapt to the host cell environment.

### LpxA T36N is less stable than wild type LpxA and has reduced enzyme activity

**Figure 7:**
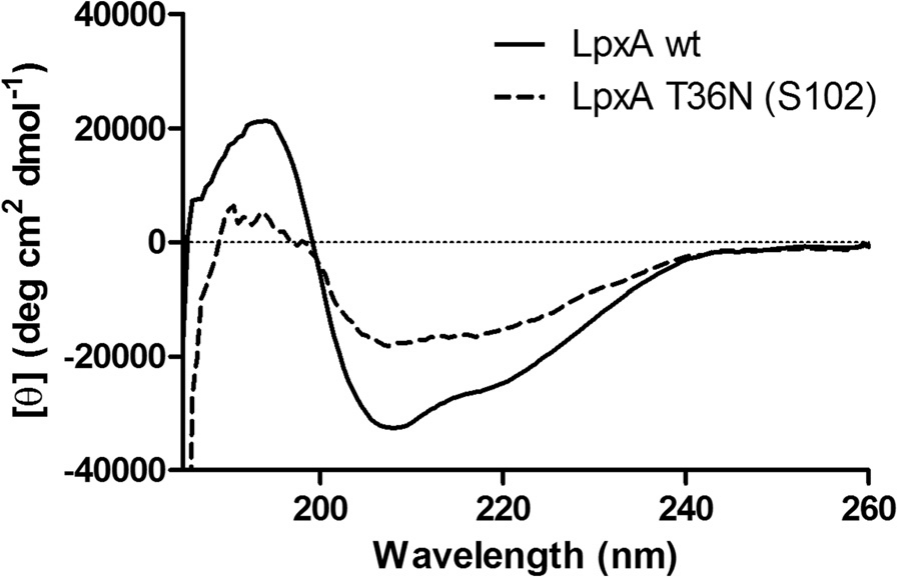
**LpxA T36N displays reduced secondary structure compared to wild type LpxA.** Circular Dichroism Spectra of Wild Type *F. tularensis* LpxA and LpxA T36N. Molar ellipticity curve generated from circular dichroism spectra for wild type LpxA at 125 μg ml^−1^, 100 μg ml^−1^, and 65 μg ml^−1^ (solid line), and LpxA T36N at 65 μg ml^−1^ (dotted line). The graph represents the mean of at least three experiments.

The suppressor mutation in LpxA T36N may be altering the structure of the protein and affecting the function of LpxA. The coils of the β-helix of LpxA are specified by hexapeptide repeats [20]. There are specific side-chain interactions in the parallel β-helices, where an asparagine ladder forms H-bonds with itself or main chain amides to stabilize the tight turn of the beta-helix [21]. Introducing a new asparagine may change the stability of the tight turn of the beta-helix. Figure 7 shows the molar ellipticity calculated from the circular dichroism (CD) spectra of LpxA wild type using three concentrations ranging from 125 μg ml^−1^ to 65 μg ml^−1^. The large maximum near 194 nm and minimum near 208 nm and 220 nm correspond with the secondary structure of LpxA representing CD spectra for α-helices, left-handed β-helices, and β-turns. The molar ellipticity values for all concentrations of wild type were the same while the values for LpxA T36N displayed reduced secondary structure (Figure 7). LpxA T36N was loaded at 65 μg ml^−1^, but spectra were present for only about 37 μg ml^−1^ (Figure 7). The CD spectrum and molar ellipticity values for LpxA T36N suggests that 56% of the protein is not folded into the correct secondary structure and is different from wild type LpxA.

**Figure 8:**
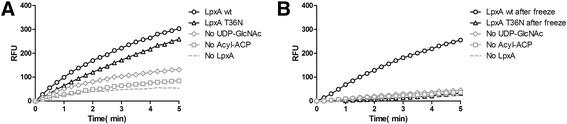
**LpxA T36N is less stable and less active than wild type LpxA.** Continuous Fluorescence Enzyme Assay with Wild Type *F. tularensis* LpxA and LpxA T36N. **(A)** Graphs of the complete LpxA enzyme assay with wild type *F. tularensis* LpxA-His (dark green), LpxA-His T36N (dotted blue), and control reactions without, either nucleotide (purple), acyl-ACP (light green), or acyltransferase (black). **(B)** Graphs of the complete LpxA reaction after LpxA samples were frozen at −80°C. Wild type *F. tularensis* LpxA-His (dark green), LpxA-His T36N (dotted blue), and control reactions without, either nucleotide (purple), acyl-ACP (light green), or acyltransferase (black). The complete LpxA mixture contained 20 mM Hepes (pH 8.0), 40 μM 3-hydroxypalmitoyl-ACP, 4 mM UDP-GlcNAc, 10 nM *F. tularensis* LpxA-His_6_ (added 5 minutes after ThioGlo) in a final volume of 100ul. The reaction was incubated at 25°C, and its progress was monitored continuously at λex = 379 nm and λem = 513 nm for 10 minutes at 15 second intervals. Control reactions were run in a similar fashion with the omission of substrate or enzyme as indicated. Each graph is a representative of three experiments.

To determine if the T36N mutation in LpxA altered enzyme activity, we used a continuous fluorescence enzyme assay for LpxA, which measures the acyl group transfer catalyzed by LpxA (Figure 8) [22]. The assay monitors the fluorescence of ThioGlo 1(methyl 10-(2,5-dioxo-2,5-dihydro-1H-pyrrol-1-yl)-9-methoxy-3-oxo-3H-benzo[f]chromene-2-carboxylate), which binds to ACP once the acyl group is transferred to LpxA. Individual components were omitted as negative controls to demonstrate background levels of fluorescence. Initial velocities were calculated using linear regression analysis during the first two minutes of the assay (Table 2). The initial rate of LpxA T36N activity was 33.7% lower than the initial rate of wild type LpxA from *F. tularensis* suggesting the missense T36N mutation influences the enzyme activity of LpxA (Figure 8A). In addition, we observed significant decreases in LpxA T36N enzyme activity when compared to wild type LpxA after freezing the protein at −80°C for as short as 5 minutes, supporting the hypothesis that the LpxA T36N mutation reduces protein stability and also decreases enzyme activity (Figure 8B). The initial rate of LpxA activity for wild type was only reduced by 7.4% when frozen at −80°C, while the initial rate of LpxA T36N activity dropped by 88.5% (Table 2).

**Table 2:** **Initial rate of LpxA-catalyzed acyl group transfer**

**Name**	**Initial rate (μM/sec)**	**Statistical significance**
LpxA wt	0.6648 ± 0.179	*P < 0.05
LpxA T36N	0.4408 ± 0.103	
LpxA wt frozen	0.6155 ± 0.263	***P < 0.0001
LpxA T36N frozen	0.0509 ± 0.0196	

Statistical significance was determined using Student’s t test comparing LpxA wt to LpxA T36N (*, P <0.05) and LpxA wt frozen to LpxA T36N frozen (***, P <0.0001).

These data demonstrate that the suppressor LpxA T36N is less stable and less active than wild type LpxA. In addition, the induced expression of *lpxA* in the absence of *ripA* is detrimental to bacterial growth. Together, these findings support the conclusion that the amount of LpxA protein is modulated by *F. tularensis* in a RipA dependent manner, and this control is important for adaptation to the intracellular environment.

## Conclusions

The main goal of this study was to ascribe biological function to RipA, a key factor for *F. tularensis* intracellular growth, by isolating, mapping and characterizing an extragenic mutant S102. S102 and 4 other independently derived extragenic mutations mapped to *lpxA* or *glmU*. Both enzymes (LpxA and GlmU) are essential components of the lipid A biosynthesis pathway. By taking a multidisciplinary approach, combining genetics and biochemistry, we determined RipA influences LpxA stability in *F. tularensis*. What remains to be determined is the role RipA plays during intracellular growth, and how LpxA and RipA prime the bacteria to survive and withstand the host defenses that usually kill invading pathogens. Understanding RipA function provides an important insight into the specific mechanisms used by *F. tularensis* to modulate LpxA—the first enzyme in the lipid A biosynthesis pathway.

The suppressor mutation in S102 made LpxA less stable, and the induced expression of wild type *lpxA* in the absence of *ripA* inhibited bacterial growth, supporting the conclusion that the interaction between RipA and LpxA is critical for controlling the first step in the lipid A synthesis pathway of *F. tularensis*. The tight regulation of the lipid A biosynthesis pathway may be important for adapting the membrane to withstand host environmental stresses. A decrease in the activity of LpxA may stimulate the phospholipid biosynthetic pathway to divert the pool of hydroxyacyl-ACP more towards the synthesis of phosphatidylethanolamine. Homoviscous adaptation is critical for bacteria to respond to environmental changes, such as in temperature, osmolality, salinity and pH [23]. Phosphatidylethanolamine and phosphatidylglycerol are the two major phospholipids present in *F. tularensis* contributing to 21% of the dry weight of the bacteria, which is high relative to other bacteria, and may allow *F. tularensis* to resist a diverse array of host defenses [14]. Recycling of the phospholipids and sugars that are used as intermediates in the synthesis of outer membrane components is critical for bacterial bilayer stability during peak infection, and is a tightly regulated process. During intracellular growth, when long chain fatty acids are in short supply it may be critical to control the lipid A synthesis pathway so phospholipid synthesis can continue. For example, LpxC (the second step in lipid A biosynthesis) is proteolyticly regulated by FtsH to control its activity [24]. The phospholipid acyl chains determine the viscosity of the membrane; with unsaturated chains providing more membrane fluidity and long saturated chains provide more rigidity [23]. *F. tularensis* lipid A and phospholipids are composed of much longer fatty acid chains than in *E. coli*, which could in part help *F. tularensis* adapt to the intracellular environment.

In conclusion, we have identified a new protein involved in modulating lipid A synthesis, which may be an important player in membrane remodeling during host cell infections. However, the precise changes in *F. tularensis* membrane composition during intracellular growth need to be further characterized.

## Methods

### Bacterial strains

All bacterial strains, plasmids, and primers used in this study are listed in Additional file 6: Table S1. *F. tularensis* subsp. *holarctica* LVS was obtained from Ft. Collins, CO. *Francisella* strains were maintained on chocolate agar supplemented with 1% IsoVitalex (BD), Chamberlains defined media (CDM) [25], or in BHI supplemented with 1% IsoVitalex at 37°C.

### Whole genome sequencing and PCR verification

Genomic DNA was purified from 10 ml cultures of *F. tularensis* grown in CDM using the MasterPure DNA purification kit (Epicentre). DNA was dissolved in 200 μl of EB buffer at 50 ng μl^−1^ and submitted for whole genome sequencing at the High-Throughput Sequencing Facility at the University of North Carolina at Chapel Hill. Using the Genome Analyzer IIx (Illumina) we produced 36 bp single-end reads that were mapped to the annotated genome on NCBI (NC_007880). By aligning the reads from the suppressor mutant strain to this reference we could identify the suppressor mutation. Alignments were made using SOAP with default parameters. Average sequence coverage was over 50 for the 1.89 Mb genome [26]. Single nucleotide polymorphisms, deletions and insertions were located using SOAP and BLAT [27]. A total of 4 mutations and 55 zero coverage regions were identified. Mutations also present in our wild type laboratory strain and the Δ*ripA* strain were discarded as background mutations, leaving one unique mutation present in S102. To verify all polymorphisms detected by Illumina sequencing we PCR amplified the regions of interest and sequenced all strains including wild type *F. tularensis*, Δ*ripA*, and all suppressors (Genewiz, Inc.). Following further confirmatory sequencing all but one mutation were eliminated as background mutations.

### Gentamicin protection assay

J774A.1 macrophage-like cells and TC-1 epithelial cells were inoculated with LVS at a multiplicity of infection (MOI) of 100. All LVS strains were grown overnight in CDM prior to inoculation. The cells were incubated with the bacterial inoculum for 2 hours (J774A.1) or 4 hours (TC-1) and then incubated with media containing 25 μg ml^−1^ gentamicin to kill extracellular bacteria. At 4 hours (J774A.1) or 6 hours (TC-1), and at 24 hours post infection medium was removed, cells were scraped from the plate, serially diluted in PBS, and plated to determine the number of viable bacteria. To enrich for extragenic suppressor mutations in Δ*ripA* the infection was extended to 36 hours before bacteria were enumerated.

### Extragenic suppressor repair using allelic exchange

The *lpxA* (FTL_0539) allele plus 100 bp of flanking DNA was PCR amplified from *F. tularensis* LVS genomic DNA. The amplified fragment was cut with BamHI and NotI and ligated into the SacB suicide vector, pMP812 [28]. Allelic exchange was achieved by transformation, selection for plasmid co-integration, and counter selection on media containing 10% sucrose. DNA sequencing confirmed replacement of the mutant allele with the wild type [29].

### Co-immunoprecipitation of LpxA and RipA

LpxA-HA was immunoprecipitated from mid-exponential phase *F. tularensis* LVS expressing *lpxA-HA* on a plasmid grown in CDM and pelleted by centrifugation at 13,000 × g for 5 minutes. Bacterial pellets were lysed using 150 mM NaCl, 1% Triton X-100, 50 mM Tris HCl (pH 8.0) and incubated for 30 minutes while shaking. Cell debris was removed by centrifugation for 5 minutes at 13,000 × g. Protein lysates were then incubated with 80 μl of μMACS™ Anti-HA MicroBeads (Miltenyi Biotec) for 30 minutes at 4°C. Following the manufacturers protocol, the proteins bound to the μMACS^TM^ Anti-HA MicroBeads (Miltenyi Biotec) were loaded onto the μ column in the magnetic field of the μMACS separator. The column was washed with 5 column volumes of 150 mM NaCl, 1% Igepal CA-630, 0.5% sodium deoxycholate, 0.1% SDS, and 50 mM Tris HCl (pH 8.0) and the protein bound to LpxA-HA were eluted using hot 50 mM Tris HCL (pH 6.8), 50 mM DTT, 1% SDS, 1 mM EDTA, 0.005% bromphenol blue, and 10% glycerol. All samples were analyzed by SDS-PAGE and Western blot. Immunoprecipitation was performed on lysates of the strain with *ripA*-HA integrated in the chromosome and *lpxA*-V5 on a plasmid (Additional file 6: Table S1, SKI22) as described above following the manufacturer’s protocol for μMACS™ Epitope Tag Protein Isolation Kit (Miltenyi Biotec).

### Membrane fractionation

Membranes were fractionated as described previously by ultracentrifugation and Sarkosyl extraction [1]. Briefly, mid-exponential phase bacteria were lysed by bead beating two times for 45 seconds using Lysing Matrix Tubes (MP Biomedicals). The lysates were clarified at max speed in a microcentrifuge for 5 minutes and crude membranes pelleted by ultracentrifugation for 2 hours at 100,000 × g. To enrich for cytoplasmic membranes from the pelleted crude membrane fraction 0.5% Sarkosyl was added overnight to selectively solubilize the inner membrane. The outer membrane was separated from the inner membrane by ultracentrifugation for 1 hour at 100,000 × g.

### Membrane purification and thin layer chromatography

*F. tularensis* 100 ml cultures were grown to optical density of 1.0 (OD_600_) or a T225 flask of J774A.1 cells were infected with *F. tularensis* at an MOI of 500. The bacteria were harvested by centrifugation and washed twice with PBS. The pellet was suspended in 8 ml of PBS. To the cell suspension 10 ml of chloroform and 20 ml of methanol were added to form a single phase (chloroform, methanol, and aqueous ratio of 1:2:0.8, v/v/v) as previously described [30]. After one hour of incubation at room temperature, the insoluble debris was removed by centrifugation at 3000 × g for 30 minutes. The supernatants were transferred to clean solvent-resistant bottles, and 10 ml of chloroform and 10 ml of PBS were added to generate a two-phase system. After mixing, the samples were centrifuged at 3000 × g for 15 minutes, and the lower phase and interface was collected. Samples were dried under a stream of nitrogen or in a speed vac. Thin layer chromatography was performed on purified membrane fractions dissolved in a total of 10 μl of chloroform and methanol (4:1) and spotted onto a silica gel 60 Å chromatography plate (Whatman). The plate was developed in the solvent chloroform, methanol, water, and acetic acid (25:15:4:2, v/v/v/v) and sprayed with 10% sulfuric acid in ethanol and charred at 100°C for 20 minutes.

### Western blots

Mini-Protean® TGX™ 4–20% precast gels (BioRad) were loaded with equal amounts of total protein from indicated samples and run at 250 V using Tris-glycine SDS running buffer. For Western blot assays, gels were transferred to nitrocellulose membranes and then blocked overnight with 1% bovine serum albumin in PBS-Tween 20. All antibodies were incubated at room temperature for 2 hours then washed with 3 volumes of PBS-Tween 20. The primary antibodies used were goat anti-RipAaa1-19, mouse anti-HA monoclonal antibody (Sigma), mouse anti-V5 monoclonal antibody (Sigma), mouse anti-*F. tularensis* LPS (US Biologicals F6070-C2), monoclonal anti-IglC (BEIresources NR-3196), monoclonal anti-IglB (BEIresources NR-3195). The secondary antibodies used were goat anti-rabbit IgG IRDye 680CW and 800CW or goat anti-mouse IgG IRDye 680CW and 800CW. Proteins were detected using near infrared fluorescence at 700 nm or 800 nm with the Odyssey Infrared Imaging System (LI-COR Biosciences).

### Lipid A purification

*F. tularensis* 100 ml cultures were grown to OD_600_ of 1.0. The cells were harvested by centrifugation at 10,000 × g for 10 minutes in a RC5C centrifuge with the GSA rotor (Sorvall Instruments). Cells were resuspended in 10 ml of TE buffer with 50 μg ml^−1^ proteinase K and incubated for one hour at 65°C. LPS was precipitated with 0.3 M sodium acetate and three volumes of 100% ethanol after incubation at −20°C for 2 hours, the LPS was centrifuged for 10 minutes at 10,000 × g as previously described [12]. The pellet was suspended in 5 ml of water and LPS was precipitated with ethanol and sodium acetate a second time. The LPS pellet was suspended in 5 ml of water with 20 μg ml^−1^ each of DNase and RNase and incubated for 2 hours at 37°C. Both 5 ml of LPS and 5 ml of phenol were warmed separately to 65°C then combined, mixed by vortexing, and incubated at 65°C for 30 minutes. LPS was then cooled on ice and centrifuged at 2000 × g for 10 minutes in an Allegra 6R centrifuge (Beckman Coulter). The aqueous layer and interface were collected. LPS was then ethanol precipitated 3× as above with 0.3 M sodium acetate and 100% ethanol. The LPS pellet was then suspended in 5 ml of 1% acetic acid and incubated at 100°C for three hours to separate the O-antigen from lipid A. Samples were then centrifuged at 14,000 × g for 30 minutes in a 5424 centrifuge (Eppendorf). The lipid A pellet was washed in water and centrifuged again for 30 minutes at 14,000 × g. The lipid A pellet was suspended in 60 μl of water. Then 50 μl of methanol, and 100 μl of chloroform was added to the lipid A pellet and centrifuged at 8,000 × g for 10 minutes. The bottom organic layer and the interface were collected.

### Positive ion mode mass spectrometry on Lipid A

Purified Lipid A samples were analyzed using a matrix-assisted laser desorption ionization fourier transform mass spectrometer (MALDI-FTMS). Spectra were obtained in a positive-ion mode using 1:1 matrix of methanol to 2,-5 dihydrozybenzoic acid (DHB). Lipid A samples were dissolved into (3:1, v/v) chloroform and methanol.

### Quantitative RT-PCR

Total RNA was isolated from mid exponential phase cultures using RiboPure-Bacteria kit (Ambion) adding TRIzol, bead beating, and then adding chloroform to separate the aqueous phase containing the RNA. DNA was removed using DNase (Promega) for 30 minutes at 37°C. Quantitative reverse transcriptase PCR (qRT-PCR) was performed in a 96-well format using the SensiFAST SYBR One-Step Kit (Bioline) following the manufacturer’s protocol. Thermocycling and detection were performed using the iCycler thermal cycler (Bio-Rad). Both a positive control using a genomic DNA ladder and a negative control with no reverse transcriptase was analyzed for each run. All *lpxA* starting quantity (SQ) values were normalized to the mean SQ values for *gyrA*. Data represent three independent experiments performed in triplicate. Significance was determined using an unpaired two-tailed *t*-test with unequal variance.

### Anhydrotetracycline inducible gene expression system for *lpxA* and *ripA*

The *E. coli-F. tularensis* shuttle vector was created with *lpxA* under the control of the *rpsLp-tetR* as previously described [19]. The *E. coli-F. tularensis* shuttle vector containing *ripA* or *lpxA* was introduced into wild type LVS and the ∆*ripA* strain via electroporation as described previously [29]. Transformants were selected on chocolate agar supplemented with 1% IsoVitalex and 200 μg ml^−1^ Hygromycin B (Hyg; Roche Applied Sciences). In *F. tularensis,* 100 ng ml^−1^ of anhydrotetracycline (ATc; Sigma-Aldrich) was used to induce expression of *lpxA* or *ripA* as previously described [19].

### Circular dichroism

Spectra from a Chirascan Plus: steady state Circular Dichroism/Fluorescence spectrometer with titration and automated temperature ramping capabilities were recorded at 25°C using 0.1 cm water-jacketed cell. LpxA samples were diluted to 3–10 μM in 10 mM potassium phosphate, pH 8.0. Spectra were measured from 260 to 190 nm. Molar ellipticity was calculated using Equation 1,

$$ \left[\theta \right] = \frac{\varDelta A}{C\ast I} $$

where ΔA is the change is CD spectra obtained from 260 to 190 nm, C is the molar concentration of LpxA, and I the cell path length. Following polarimetric conversions molar ellipticity was reported in degrees cm^2^ dmol^−1^. Spectra were measured at least three times with independent biological replicates.

### LpxA-His_6_ purification for enzyme assay

Strains of interest were inoculated into 500 ml of Luria-Bertani (LB) medium containing 100 μg ml^−1^ of Ampicillin and were incubated while shaking at 37°C until an OD_600_ of 1.0 was reached. The cultures were induced with 1 mM IPTG followed by incubation at 25°C for four hours. Cells were harvested by centrifugation at 10,000 × g for 10 minutes at 4°C, and processed immediately or frozen at −80°C. Cells were resuspended in 6 ml of 20 mM Hepes, 100 mM NaCl at pH 8.0. Cell suspensions were disrupted by French press at 20,000 psi. Cellular debris was removed by centrifugation at 14,000 × g for 5 minutes at 4°C in a 5424 centrifuge (Eppendorf). Soluble crude cytosol was loaded onto 3 ml of nickel-nitriol-triacetic acid (Ni-NTA) resin (Qiagen). The resin was washed with 10 column volumes of loading buffer containing 500 mM NaCl and then eluted with 20 mM Hepes and 500 mM imidazole (pH 8.0). Purified *F. tularensis* LpxA-His_6_ was desalted using Slide-A-Lyzer Dialysis Cassette (Thermo Scientific) and dialyzed against 20 mM Hepes pH 8.0. Protein concentrations were determined by bicinchoninic acid (BCA) assay. The purification, and acylation of holo-ACP was carried out as described previously, except hydroxypalmitic acid (C16:0) was used in place of hydroxymyrstic acid (C14:0) [22].

### Fluorescence enzyme assay for LpxA-His_6_

Following the procedure developed by Dotson and Jenkins, the assay was performed at 25°C in 20 mM Hepes (pH 8.0) at a final volume of 100 μl monitoring the conversion of ThioGlo 1(methyl 10-(2,5-dioxo-2,5-dihydro-1H-pyrrol-1-yl)-9-methoxy-3-oxo-3H-benzo[f]chromene-2-carboxylate) to ThioGlo-ACP produced in the LpxA acyltransferase-catalyzed reaction [22]. To the appropriate well 40 μM of acyl-ACP, 4 mM UDP-GlcNAc, and 10 μM ThioGlo were added. To initiate the reaction, 10 nM LpxA-His_6_ was added directly to the well and mixed gently. The SpectraMax M2*e* plate reader (Molecular Devices) was used to continuously monitored at λ_ex_ = 379 nm and λ_em_ = 513 nm for 10 minutes at 15 second intervals. Control reactions lacking individual substrate or enzyme components were performed to demonstrate that an increase in fluorescence was both enzyme and substrate dependent. To calculate initial rates linear regression plots were generated for the first two minutes of the reaction. The fluorescence enzyme assay was repeated at least three times with independent biological replicates.

## Additional files

Additional file 1: Figure S1.
**The S103 extragenic suppressor mutant phenotype and repair in J774A.1 and TC-1 cells.** Intracellular replication of wild type *F. tularensis* LVS, ∆*ripA,* S103 GlmU (E57D), and the strain containing the repaired S103 extragenic suppressor mutation (GlmU D57 → E) was assessed within J774A.1 macrophages (A) and TC-1 epithelial cells (B) using the gentamicin protection assay. Each graph is the mean of at least three independent experiments done in triplicate, and the error bars represent the standard deviation. Statistical significance was determined using Student’s *t* tests comparing the suppressor strain to the repaired strain. **P* < 0.05. Additional file 2: Figure S2.
**LpxA localization.** Membrane fractionation separating the soluble (Sol), inner membrane (IN), and outer membrane (OM) fractions using ultracentrifugation and Sarkosyl extractions. Fractions from wild type LVS with *lpxA-HA* in trans, and the ∆*ripA* strain with *lpxA-HA* in trans were probed with the N-terminal RipAaa1-19 antibody for an inner membrane control, the IglC antibody for the soluble fraction control (a small fraction of IglC was also present in the inner membrane fraction), and LPS antibody for the outer membrane control. The monoclonal anti-HA antibody was used to probe for LpxA-HA. This figure is a representative of three independent experiments. Additional file 3: Figure S3.
**Membrane and LPS composition of Suppressor S102.** (A) Thin layer chromatography of purified membrane fractions from LVS, ∆*ripA*, and S102 using the Bligh and Dyer method [24]. The plate was developed in the solvent chloroform, methanol, water, and acetic acid (25:15:4:2, v/v/v/v) and sprayed with 10% sulfuric acid in ethanol and charred at 100°C for 20 minutes. (PG) phosphatidylglycerol, (PE) phosphatidylethanolamine, (A1) Lipid A (B) From left to right, thin layer chromatography of purified membrane fractions from J774A.1 macrophages, J774A.1 macrophages infection with LVS wild type, and J774A.1 macrophages infection with ∆*ripA* using the same Bligh and Dyer method. (*) indicate unique lipids identified during infection. (C) LPS Western blots on whole cell lysates from LVS, ∆*ripA*, and S102. The blot was probed with *Francisella tularensis*, LPS antibody. All experiments shown here are representatives, and were repeated at least three times. Additional file 4: Figure S4.
**LPS quantity.** Quantity of LPS purified from 100ml cultures of LVS, Δ*ripA*, and S102 at a Klett 100, using the purpald assay. Following the procedure previously developed, 50 μl of LPS diluted 1/10 in water was added to a 96 well plate followed by 50 μl of 32 mM periodate (NaIO_4_) [18]. The mixture was then incubated for 25 minutes at room temperature followed by the addition of 50 μl of 136 mM purpald reagent dissolved in 2N NaOH. The reaction was incubated for 20 minutes followed by the addition of 50 μl of NaIO_4_. After 20 minutes of incubation at room temperature the foam was removed with 20 μl of 2-propanol. The reaction was quantified by reading the absorbance at formaldehyde generated at 550 nm on the Magellan M200 Tecan. LPS concentrations were calculated based on the *E. coli* LPS standard curve. The graph represents three independent experiments and error bars represent the standard deviation. Additional file 5: Figure S5.
**Mass spectrometry on Lipid A.** Lipid A samples were analyzed using a matrix-assisted laser desorption ionization Fourier transform mass spectrometer (MALDI- FTMS). Spectra were obtained in a positive-ion mode. (A) Wild type LVS, (B) ∆*ripA*, and (C) S102 each have a peak at m/z 1549 and m/z 1521, which corresponds to the intact lipid A molecule with a 1′ phosphate and a 3′ stearoyl or palmitoyl acyl chain, respectively. All graphs are representative of at least three independent experiments. Additional file 6: Table S1.
**Bacterial strains, plasmids, and primers [** 31 **].**
Additional file 7: Table S2.
**Zero coverage of whole genome sequencing.**
