# Supplementary figures and images for: Extragenic suppressor mutations in ΔripA disrupt stability and function of LpxA
Source: BMC Microbiol. 2014 Dec 31;14:336. doi: 10.1186/s12866-014-0336-x (PMC4322802; doi:10.1186/s12866-014-0336-x)

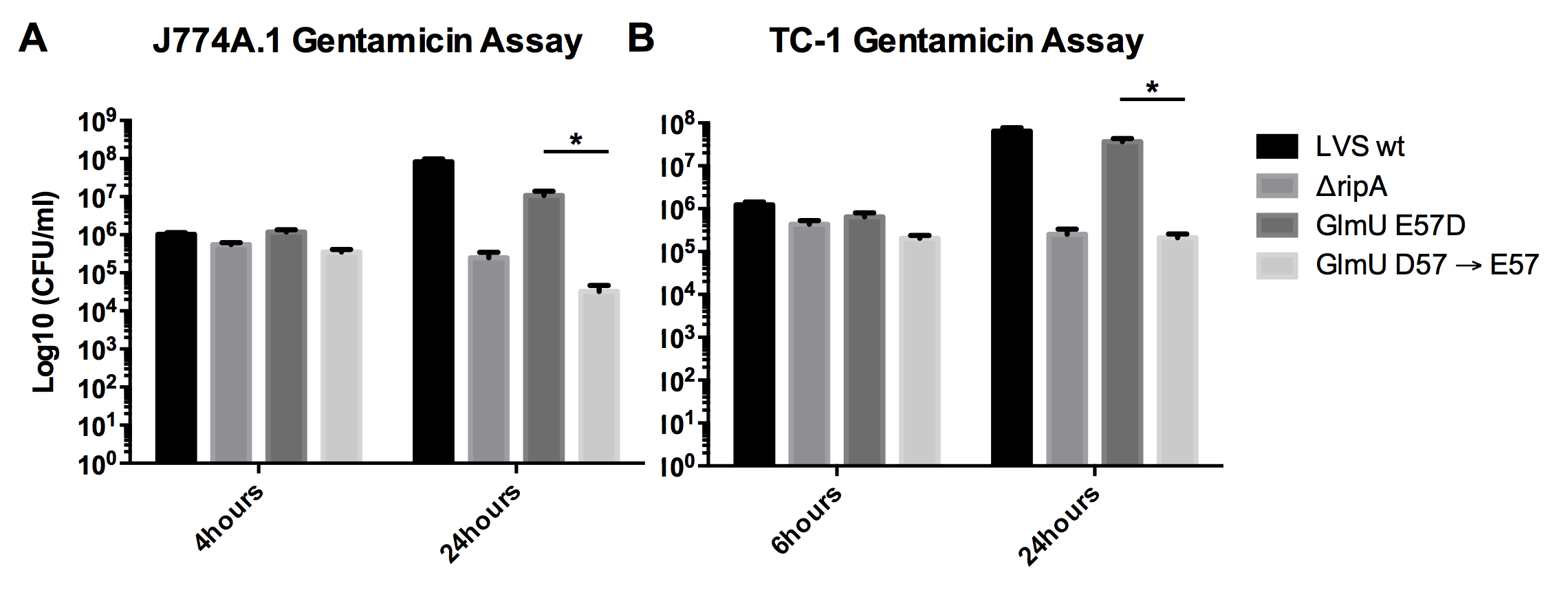

Supplement: Additional file 1: Figure S1. — The S103 extragenic suppressor mutant phenotype and repair in J774A.1 and TC-1 cells. Intracellular replication of wild type F. tularensis LVS, ∆ripA, S103 GlmU (E57D), and the strain containing the repaired S103 extragenic suppressor mutation (GlmU D57 → E) was assessed within J774A.1 macrophages (A) and TC-1 epithelial cells (B) using the gentamicin protection assay. Each graph is the mean of at least three independent experiments done in triplicate, and the error bars represent the standard deviation. Statistical significance was determined using Student’s t tests comparing the suppressor strain to the repaired strain. *P < 0.05. [file 12866_2014_336_MOESM1_ESM.tiff]

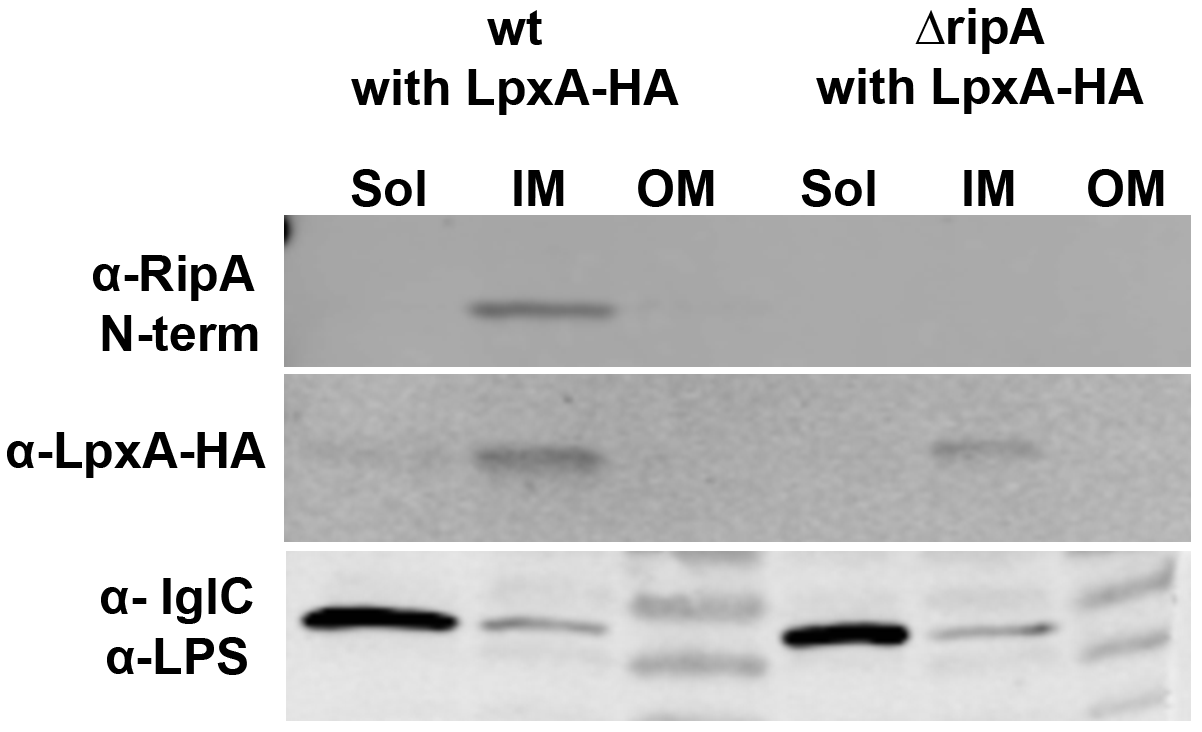

Supplement: Additional file 2: Figure S2. — LpxA localization. Membrane fractionation separating the soluble (Sol), inner membrane (IN), and outer membrane (OM) fractions using ultracentrifugation and Sarkosyl extractions. Fractions from wild type LVS with lpxA-HA in trans, and the ∆ripA strain with lpxA-HA in trans were probed with the N-terminal RipAaa1-19 antibody for an inner membrane control, the IglC antibody for the soluble fraction control (a small fraction of IglC was also present in the inner membrane fraction), and LPS antibody for the outer membrane control. The monoclonal anti-HA antibody was used to probe for LpxA-HA. This figure is a representative of three independent experiments. [file 12866_2014_336_MOESM2_ESM.tiff]

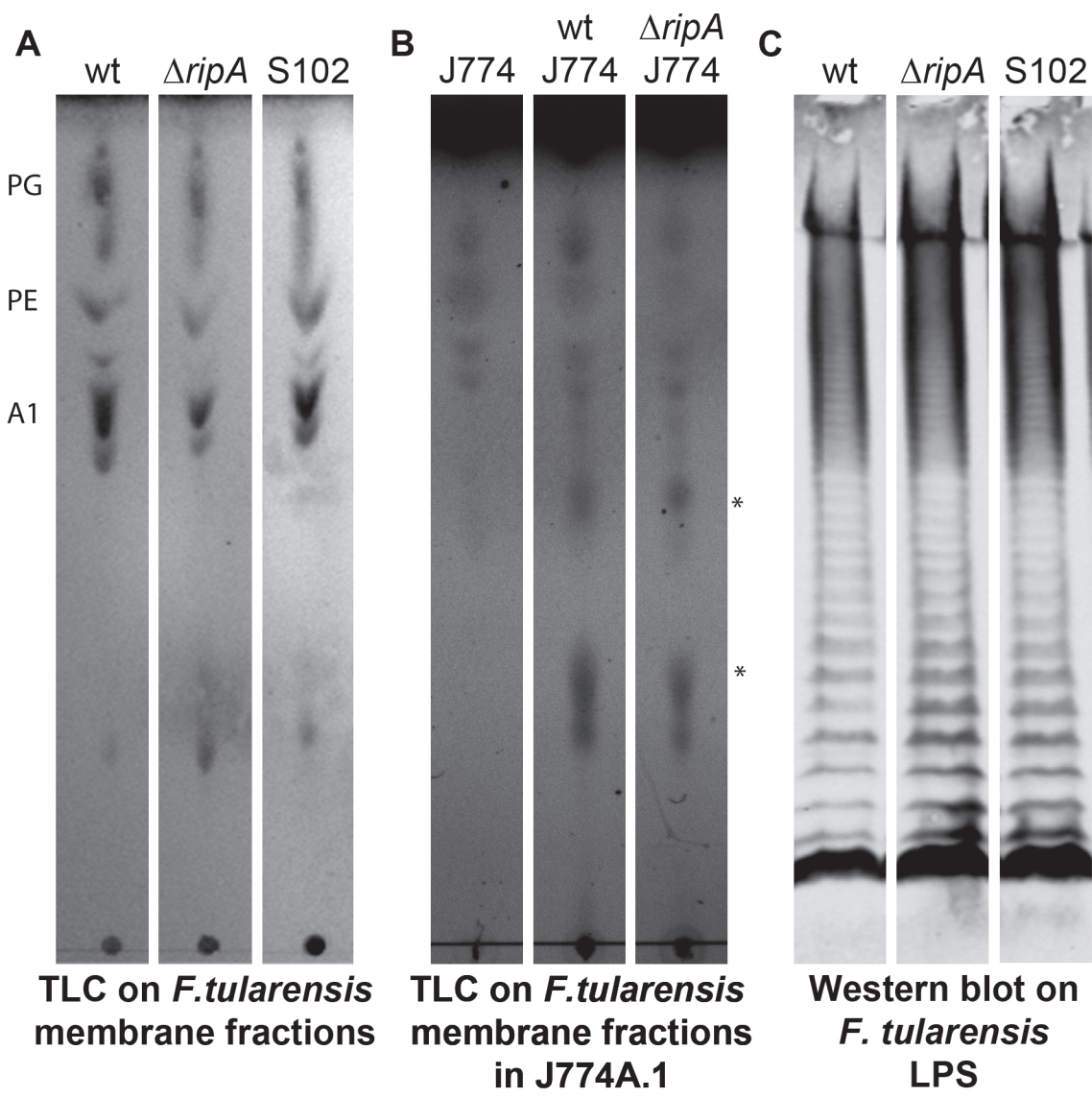

Supplement: Additional file 3: Figure S3. — Membrane and LPS composition of Suppressor S102. (A) Thin layer chromatography of purified membrane fractions from LVS, ∆ripA, and S102 using the Bligh and Dyer method [24]. The plate was developed in the solvent chloroform, methanol, water, and acetic acid (25:15:4:2, v/v/v/v) and sprayed with 10% sulfuric acid in ethanol and charred at 100°C for 20 minutes. (PG) phosphatidylglycerol, (PE) phosphatidylethanolamine, (A1) Lipid A (B) From left to right, thin layer chromatography of purified membrane fractions from J774A.1 macrophages, J774A.1 macrophages infection with LVS wild type, and J774A.1 macrophages infection with ∆ripA using the same Bligh and Dyer method. (*) indicate unique lipids identified during infection. (C) LPS Western blots on whole cell lysates from LVS, ∆ripA, and S102. The blot was probed with Francisella tularensis, LPS antibody. All experiments shown here are representatives, and were repeated at least three times. [file 12866_2014_336_MOESM3_ESM.pdf]

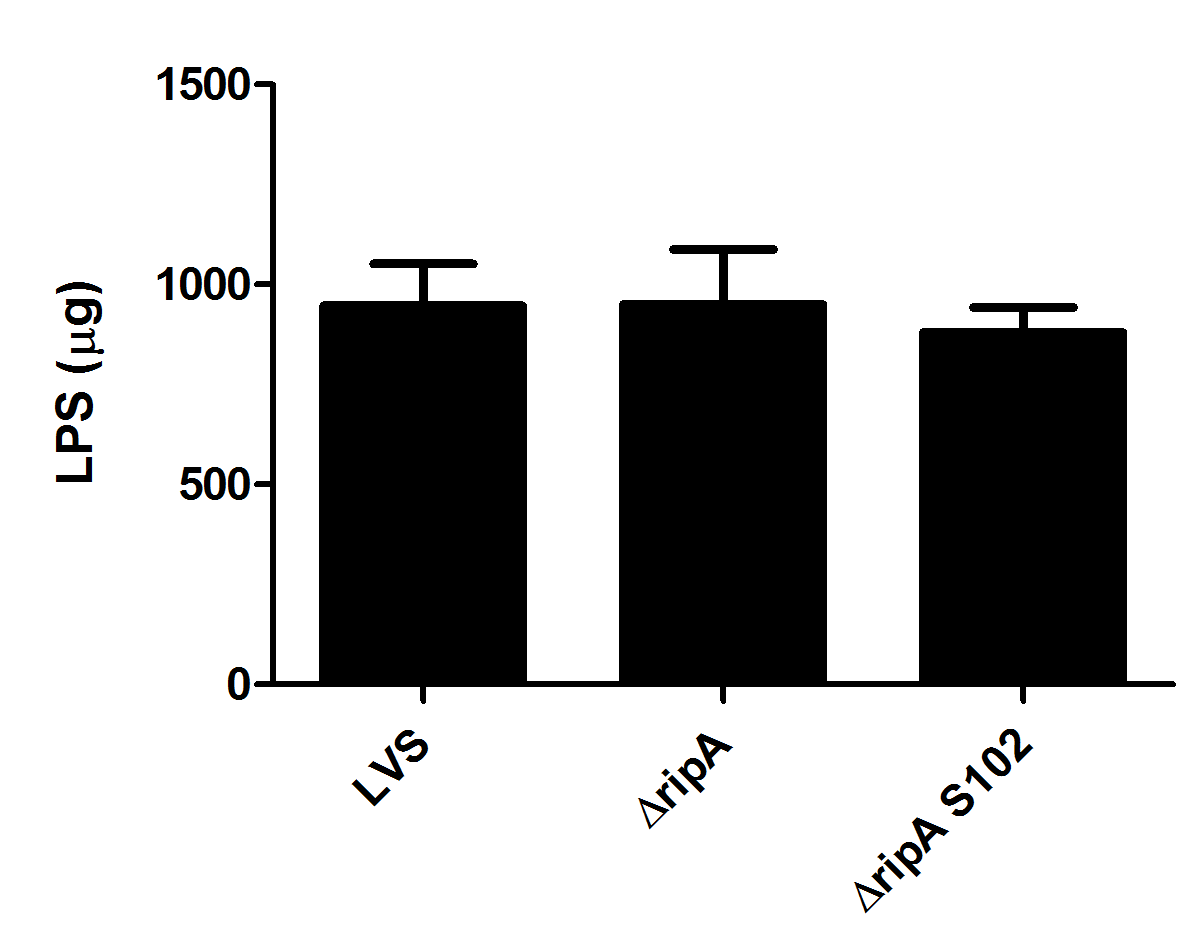

Supplement: Additional file 4: Figure S4. — LPS quantity. Quantity of LPS purified from 100ml cultures of LVS, ΔripA, and S102 at a Klett 100, using the purpald assay. Following the procedure previously developed, 50 μl of LPS diluted 1/10 in water was added to a 96 well plate followed by 50 μl of 32 mM periodate (NaIO4) [18]. The mixture was then incubated for 25 minutes at room temperature followed by the addition of 50 μl of 136 mM purpald reagent dissolved in 2N NaOH. The reaction was incubated for 20 minutes followed by the addition of 50 μl of NaIO4. After 20 minutes of incubation at room temperature the foam was removed with 20 μl of 2-propanol. The reaction was quantified by reading the absorbance at formaldehyde generated at 550 nm on the Magellan M200 Tecan. LPS concentrations were calculated based on the E. coli LPS standard curve. The graph represents three independent experiments and error bars represent the standard deviation. [file 12866_2014_336_MOESM4_ESM.tiff]

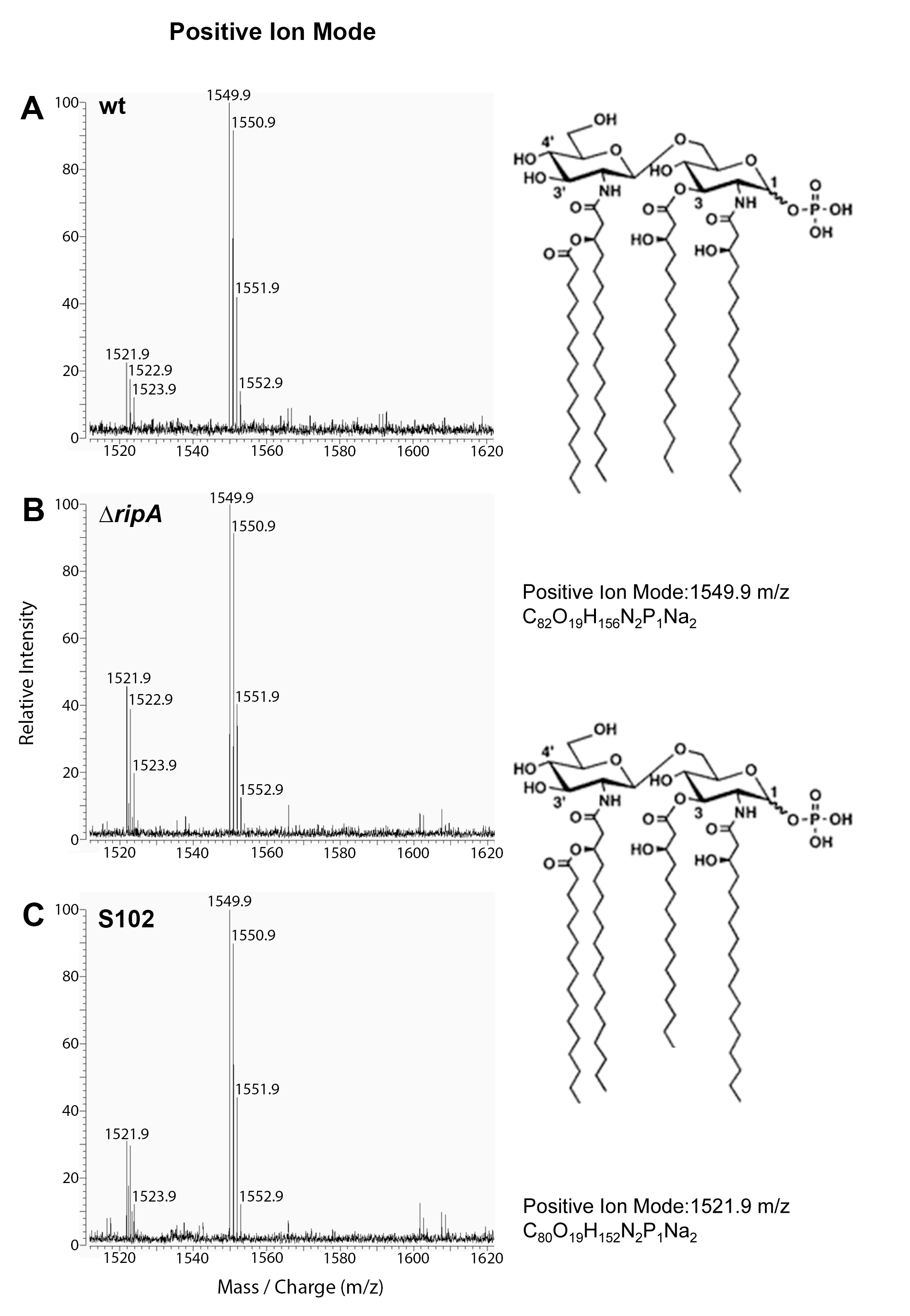

Supplement: Additional file 5: Figure S5. — Mass spectrometry on Lipid A. Lipid A samples were analyzed using a matrix-assisted laser desorption ionization Fourier transform mass spectrometer (MALDI- FTMS). Spectra were obtained in a positive-ion mode. (A) Wild type LVS, (B) ∆ripA, and (C) S102 each have a peak at m/z 1549 and m/z 1521, which corresponds to the intact lipid A molecule with a 1′ phosphate and a 3′ stearoyl or palmitoyl acyl chain, respectively. All graphs are representative of at least three independent experiments. [file 12866_2014_336_MOESM5_ESM.tiff]
