# Supplementary material for: Extragenic suppressor mutations in ΔripA disrupt stability and function of LpxA
Source: BMC Microbiol. 2014 Dec 31;14:336. doi: 10.1186/s12866-014-0336-x (PMC4322802; doi:10.1186/s12866-014-0336-x)
Supplement: Additional file 6: Table S1. — Bacterial strains, plasmids, and primers [ 31 ]. [file 12866_2014_336_MOESM6_ESM.docx]

Table S1

**Table S1**. **Bacterial Strains, Plasmids, and Primers**

| **Strain, Plasmid, or Primer** | **Genotype, Phenotype, or Sequence** | **Source** |
| --- | --- | --- |
| **Bacterial Strains** |  |  |
| *E. coli* DH10B | *E. coli* F-*mcr*A Δ(*mrr*-*hsd*RMS-*mcr*BC) φ80*lac*ZΔM15 Δ*lac*X74 *rec*A1*end*A1 *ara*D139 Δ(*ara leu*) 7697 *gal*U *gal*K *rps*L *nup*G λ- | Invitrogen |
| *E. coli* Top10 | *E. coli* F-*mcrA* Δ(*mrr-hsd*RMS-*mcr*BC) φ80*lac*ZΔM15 Δ*lac*Χ74 *rec*A1 *ara*D139 Δ*(ara-leu*) 7697 *gal*U *gal*K *rps*L (StrR) *end*A1 *nup*G λ- | Invitrogen |
| *E. coli* DH5 α | *E. coli* φ80*lac*ZΔM15 Δ(*lac*ZYA*-arg*F*)*U169 *rec*A1 *end*A1 *hsd*R17*(*r_K_*^-^,* m_K_*^+^) pho*A *sup*E44 *thi-1 gyr*A96 *rel*A1 *λ^-^* | Invitrogen |
| *E. coli* BL21(DE3) pLysS | *E.coli* F*^-^, omp*T*, hsd*S*_B_ (*r_B_*-,* m_B_*-), dcm, gal, λ(DE3), pLysS, Cm^r^* | Promega |
| *F. holarctica* LVS | *Francisella tularensis* subsp. *holarctica* LVS (taxid:376619) | CDC |
| ∆*ripA* | *Francisella tularensis* subsp. *holarctica* LVS | ([1](#_ENREF_1)) |
| RipA-HA | *Francisella tularensis* subsp. *holarctica* LVS with RipA-HA chromosomally integrated | ([2](#_ENREF_4)) |
| SKI12 | *E.coli* BL21(DE3) pLysS with LpxA-His_6_ from *F. tularensis* LVS | This work |
| SKI13 | *E.coli* BL21(DE3) pLysS with LpxA-His_6_ D144N from F. tularensis LVS S101 | This work |
| SKI14 | *E.coli* BL21(DE3) pLysS with LpxA-His_6_ T36N from *F. tularensis* LVS S102 | This work |
| SKI15 | *E.coli* BL21(DE3) pLysS with RipA-His_6_ from *F. tularensis* LVS | This work |
| SKI16 | *Francisella tularensis* subsp. *holarctica* LVS with LpxA T36N | This work |
| SKI17 | *Francisella tularensis* subsp. *holarctica* LVS with LpxA N36🡪T | This work |
| SKI18 | *Francisella tularensis* subsp. *holarctica* LVS containing pSKI07 | This work |
| SKI19 | *Francisella tularensis* subsp. *holarctica* LVS ∆*ripA* containing pSKI07 | This work |
| SKI20 | *Francisella tularensis* subsp. *holarctica* LVS containing pSKI08 | This work |
| SKI21 | *Francisella tularensis* subsp. *holarctica* LVS ∆*ripA* containing pSKI08 | This work |
| SKI22 | *Francisella tularensis* subsp. *holarctica* LVS containing pSKI09 | This work |
| SKI23 | *Francisella tularensis* subsp. *holarctica* LVS containing pEDL20 | (19) |
| SKI24 | *Francisella tularensis* subsp. *holarctica* LVS ∆*ripA* containing pEDL20 | (19) |
| SKI25 | *Francisella tularensis* subsp. *holarctica* LVS ∆*ripA* containing pSKI09 | This work |
| SKI26 | *Francisella tularensis* subsp. *holarctica* LVS with RipA-HA chromosomally integrated with pSKI10 | This work |
| SKI27 | *Francisella tularensis* subsp. *holarctica* LVS with pSKI10 | This work |
| **Plasmids** |  |  |
| pSKI05 | pET23a+*lpxA*-His_6_ from *F. tularensis* LVS AmpR | This work |
| pSKI06 | pET23a+*lpxA*-His_6_ T36N from *F. tularensis* LVS S102 AmpR | This work |
| pSKI07 | pMP822 with LpxA-HA HygR *blaB* promoter | This work |
| pSKI08 | pMP822 with LpxA-HA T36N HygR *blaB* promoter | This work |
| pSKI09 | *FTRp-lpxA, rpsLp-tetR,* HygR | This work |
| pSKI10 | *FTRp-lpxA-V5, rpsLp-tetR,* HygR | This work |
| pSKI11 | pMP812 with wt *F. tularensis lpxA* | This work |
| pEDL20 | *FTRp-ripA, rpsLp-tetR,* HygR | (19) |
| pET23a+ | N-terminal T7-Tag / C-terminal His-Tag with T7 promoter | (22) |
| pMP812 | sacB suicide vector, KanR, SucS | (28) |
| pMP590 | *sacB* suicide vector, KmR, SucS | (29) |
| pMP822 | *E.coli-F.tularensis* shuttle vector, HygR *blaB* promoter | (31) |
| pMP831 | *E.coli-F. tularensis* shuttle vector, HygR | (28) |
| pEDL50 | Conjugative *sacB* suicide vector, HygR, SucS | (19) |
| **Primers** | 5’🡪3’ |  |
| Sequencing Validation | |  |
| FTL_0146F | ggatgagcctttttctgcac | This work |
| FTL_0146R | gcataacgagcccagtcaat | This work |
| FTL_0717F | ggcgaagaaacaagaattgc | This work |
| FTL_0717R | atgacgatagaaccgccaga | This work |
| FTL_1388F | gcaggtgtagttgctgctattg | This work |
| FTL_1388R | atcatcgcactgccattacc | This work |
| FTL_0453F | tatcagcagcagcaacttgg | This work |
| FTL_0453R | ccaactattgcaccttcacg | This work |
| FTL1914F | atccggcgaagatttcatta | This work |
| FTL1914R | tgaggtaaccaagcaatttcg | This work |
| FTL_0539F | tgttgagaaagctggtggtg | This work |
| FTL_0539R | ttcgcgaagtaccaatcaca | This work |
| Chromosomal Gene Disruption and Vector Cloning | | |
| LpxA F | ggcggtccgctaatcgtgatacatagtttggcagtagtacatgag | This work |
| LpxA-HA R | cccctcgag**ttatccaccaccagcataatctggaacatcataaggatagccacc**tcttagtatacctcttcgcgaagtac | This work |
| LpxA-V5 R | gggacccggg**ttatccagtagaatctagtcctagtagtgggtttggtattggttttcc**tcctcttagtatacctcttcgcgaagtac | This work |
| LpxA F pET23 | cagcatatgctaatcgtgatacatagtttggcagtagtacatgag | This work |
| LpxA R  pET23 | cccctcgagtcttagtatacctcttcgcgaagtacc | This work |
| Real Time PCR | |  |
| gyrA RT F | ctatacgctagtagatggacaaggtaactt | This work |
| gyrA RT R | ctatacgctagtagatggacaaggtaactt | This work |
| lpxA LVS F | gtcgtggatttacgcctgaagag | This work |
| lpxA LVS R | tcttcttttgccatcgctttg | This work |

Underlined text indicates restriction sites

Bold and underlined text indicates His-tag, HA-tag, or V5 tag
